# A momentary assessment study on emotional and biological stress in adult males and females with autism spectrum disorder

**DOI:** 10.1038/s41598-021-93159-y

**Published:** 2021-07-08

**Authors:** Kim van der Linden, Claudia Simons, Wolfgang Viechtbauer, Emmy Ottenheijm, Thérèse van Amelsvoort, Machteld Marcelis

**Affiliations:** 1 GGzE, Mental Health Institute Eindhoven, P.O. Box 909, 5600AX Eindhoven, The Netherlands; 2 Department of Psychiatry and Neuropsychology, School for Mental Health and Neuroscience (MHeNS), Maastricht University, Maastricht, The Netherlands

**Keywords:** Stress and resilience, Autism spectrum disorders

## Abstract

Prospective momentary psychological and biological measures of real-time daily life stress experiences have been examined in several psychiatric disorders, but not in adults with an autism spectrum disorder (ASD). The current electronic self-monitoring study examined associations between momentary daily life stressors and (i) negative affect (NA; emotional stress reactivity) and (ii) cortisol levels (biological stress reactivity) in males and females with ASD (N = 50) and without ASD (N = 51). The Experience Sampling Method, including saliva sampling, was used to measure three types of daily life stress (activity-related, event-related, and social stress), NA, and cortisol. Multilevel regression analyses demonstrated significant interactions between group and stress (i.e., activity-related and event-related stress) in the model of NA, indicating stronger emotional stress reactivity in the ASD than in the control group. In the model of cortisol, none of the group × stress interactions were significant. Male/female sex had no moderating effect on either emotional or biological stress reactivity. In conclusion, adults with ASD showed a stronger emotional stress (but not cortisol) reactivity in response to unpleasant daily life events and activities. The findings highlight the feasibility of electronic self-monitoring in individuals with ASD, which may contribute to the development of more personalized stress-management approaches.

## Introduction

Observational and experimental stress studies report increased emotional stress levels in adults with an autism spectrum disorder (ASD) with respect to controls^1,2^. However, there is no intensive time-series data on real-life, real-world momentary emotional stress reactivity derived from individuals with ASD. Emotional stress reactivity, defined as the effect of subjective appraisals of everyday stressors on negative affect (NA) can be studied via ecological momentary assessment (EMA) and has been used in a wide range of psychiatric disorders. For example, an increased emotional stress reactivity has been found in individuals at high risk for psychosis^3^, with psychotic illness^4^, and remitted bipolar disorder^5^ compared to the general population.

Evidence also shows that dysregulation of the hypothalamic-pituitary-adrenocortical (HPA) axis may play a role in the altered stress processing of individuals with ASD, indicating that the stress hormone cortisol may be disturbed. However, reports on cortisol outcome measures are not very consistent. Both increased^6,7^, decreased^8,9^, or equal^10,11^ cortisol responses to social stressors have been found in children and adolescents with ASD compared to non-ASD individuals. To our knowledge, only two experimental studies have investigated the cortisol response in adults with ASD; both studies found a comparable cortisol response to a social stressor in the ASD and control group^1,12^. Because of this inconsistent pattern and the artificial nature of laboratory settings, studying cortisol response in a naturalistic environment may shed new light on the relationship between stress and cortisol in ASD. In the past decades, EMA studies investigated biological stress reactivity by studying associations between minor daily life stressors and momentary cortisol levels. For example, an increased cortisol response associated with daily stressors was demonstrated in 556 females in the general population^13^. In addition, studies on psychiatric samples showed an increased cortisol response to daily stressors in participants with above-average risk for psychosis^14^ and a blunted cortisol response in participants with 22q11.2 deletion syndrome^15^ relative to controls.

Not only cortisol dysregulation per se is important, but also its role in the pathway to mood disturbance. Multiple lines of research have explored the pathways involved in the stress response; the most replicated finding in the literature is that cortisol mediates the association between stress and mood in humans^16,17^ or behavior in animals^18,19^. Although this pathway has not yet been explored in adults with ASD, studies using animal models in ASD (i.e., BTRB mice) showed evidence for a mediating effect of stress hormones in the behavioral response to stressors^20,21^. The current study will, for the first time, explore whether momentary cortisol mediates emotional stress reactivity in daily life.

In addition to the above-mentioned understudied themes, sex differences in emotional and biological stress response in individuals with ASD is another area of neglect. It is well-known that stress-related disorders (e.g., depression) are twice as prevalent in females than males^22,23^ and it also has been shown that the HPA axis is particularly influenced by female sex hormones^24^. Indeed, a recent observational study demonstrated higher levels of perceived stressful life events in adult females with ASD relative to males^25^. Of note, using EMA in patients with psychosis, an increased momentary emotional stress response (i.e., increased NA) to daily stress was found in females than in males^26^. This has led to the idea that the affective pathway to psychosis may be more dominant in females, whereas the developmental pathway to psychosis may be more prominent in males. Due to a neurobiological, phenomenological, and genetic overlap between the autism and psychosis spectrum^27,28^, greater emotional, and possibly biological, stress reactivity in females could be expected in individuals with ASD. Studying sex differences in momentary stress reactivity is pivotal as it may indicate a sex-dependent underlying vulnerability to develop mood and anxiety symptoms^29,30^, knowledge that is vital for the development of tailored-treatment.

This is the first study investigating momentary emotional and biological stress reactivity in the natural flow of daily life in adults with ASD. This was done with the Experience Sampling Method (ESM), an EMA tool. The ESM is a valid and reliable method^31^ in which short questionnaires about momentary experiences are presented to participants at random moments in time. This method is less susceptible to bias and has been applied to a wide range of psychiatric disorders^32^. Although ESM has only been used in a few studies on ASD, the feasibility and usefulness of this method have been supported in this population^33,34^. For the present study, we used three valid ESM stress measures^35^, i.e., activity-related stress, event-related stress, and social stress, to measure emotional stress reactivity^3,36,37^. In addition, to assess cortisol fluctuations during the day, momentary cortisol sampling was also integrated as previously described^13–15^.

Altogether, this study examined associations between momentary daily life stressors and (i) NA (emotional stress reactivity) and (ii) cortisol (biological stress reactivity) and compared these associations across groups and sex. It was hypothesized that adults with ASD would experience greater emotional and biological stress reactivity, particularly in females, relative to controls. Second, an exploratory analysis was done to investigate whether cortisol had a mediating effect on emotional stress reactivity.

## Results

### Sample characteristics

The total sample consisted of 50 adults with ASD and 51 controls (*N* = 101 participants). None of the participants were excluded. Sociodemographic and clinical characteristics are presented in Table 1. The groups did not differ on estimated IQ (*p* = 0.636) and sex (*p* = 0.918). However, the mean age was significantly higher in adults with ASD relative to controls (*p* = 0.028). Participants completed 7861 valid ESM reports. Adults with ASD filled out more ESM reports but the difference between groups was not significant (*p* = 0.116).

**Table 1:** Sociodemographic and clinical characteristics of the research sample.

	ASD (N = 50)	Controls (N = 51)
**Age, mean (SD), range**	41.1 (12.9), 18–64	35.5 (12.2), 18–63
**Sex (m/f)**	26/24	26/25
**Civil status, *n* (%)**		
Never married	25 (50%)	14 (27%)
Married	13 (26%)	16 (31%)
Living together	3 (6%)	14 (27%)
Divorced	8 (16%)	6 (12%)
Widowed	1 (2%)	1 (2%)
**Work situation, *n* (%)**		
Household	1 (2%)	1 (2%)
School/education	4 (8%)	11 (21.5%)
Regular work full-time	6 (12%)	22 (43%)
Regular work part-time	13 (26%)	11 (21.5%)
Structured work	10 (20%)	4 (8%)
Non-structured activities	15 (30%)	1 (2%)
Other	1 (2%)	1 (2%)
**Educational level, *n* (%)**		
Primary school	1 (2%)	0 (0%)
Secondary school	12 (24%)	6 (12%)
Higher education	37 (74%)	45 (88%)
**ADOS-2 classification, *n* (%)**		
Autism	32 (64%)	
Autism spectrum	18 (36%)	
AQ score, mean (SD), range		9.4 (4.9), 0–25
**WAIS-IV subtests, mean (SD), range**		
Matrix reasoning	10.9 (2.6), 6–18	10.9 (2.2), 5–15
Vocabulary	11.8 (2.9), 5–16	11.4 (3.0), 6–19
Estimated IQ, mean (SD), range	110.1 (17.7), 79–147	108.5 (15.4), 73–141
**DSM-IV axis diagnosis, *n (%)***		
Depression current	3 (6%)	0^a^
Depression lifetime	23 (46%)	6 (12%)
**Medication use, *n***		
Antipsychotics	6	0
Antidepressants	11	3
Anxiety medications	6	0
Insomnia medications	4	0
Oral contraceptives	3	5
**Valid ESM beeps, mean (SD), range**	79.8 (12.7), 49–103	75.8 (12.9), 32–97

ASD, Autism spectrum disorder; ADOS-2, Autism Diagnostic Observation Schedule II; AQ, the Autism Spectrum Quotient; WAIS-IV, Wechsler Adult Intelligence Scale—Fourth Edition; IQ, intelligence quotient; ESM, Experience Sampling Method.

^a^Current depression was an exclusion criterion in the control group.

### Group differences in ESM measures

The ASD group reported significantly higher levels of NA (B = 0.83, *p* < 0.001), activity-related (B = 0.61, *p* < 0.001), event-related (B = 0.09, *p* = 0.028), and social stress (B = 1.21, *p* < 0.001) than controls. There was no group difference in cortisol levels (B = 0.02, *p* = 0.760).

### Group and sex differences in emotional and biological stress reactivity

#### Emotional stress reactivity

**Figure 1:**
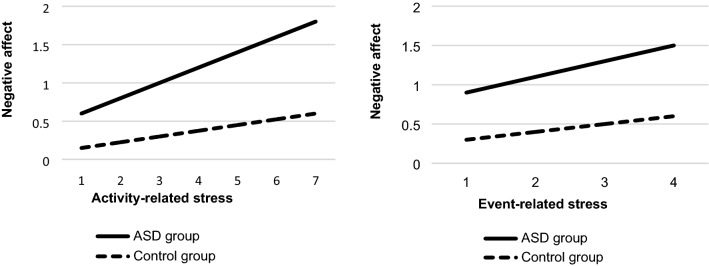
Associations between activity-related or event-related stress scores and negative affect. ASD, Autism spectrum disorder.

None of the three-way interactions were significant. As shown in Table 2, significant two-way interactions were found between group and activity-related stress or event-related stress in the model of NA. The simple slope analyses showed stronger positive associations between activity-related stress or event-related stress and NA in the ASD group relative to controls (Table 3, Fig. 1). No significant social stress × group interaction was found.

**Table 2:** Multilevel regressions estimate of stress, group, sex, and their interactions in the model of negative affect.

	Obs	B	SE	P	95% CI
Activity-related stress	7842	0.09	0.03	0.002	[0.03, 0.15]
Group		0.46	0.15	0.003	[0.16, 0.75]
Group × activity-related stress		0.10	0.04	0.012	[0.02, 0.19]
Sex		− 0.01	0.14	0.946	[− 0.29, 0.27]
Sex × activity-related stress		0.01	0.04	0.750	[− 0.07, 0.10]
Sex × group		− 0.06	0.20	0.767	[− 0.46, 0.34]
Group × sex × activity-related stress		0.02	0.06	0.683	[− 0.09, 0.14]
Event-related stress	7834	0.11	0.03	0.001	[0.04, 0.17]
Group		0.58	0.20	0.004	[0.19, 0.97]
Group × event-related stress		0.13	0.05	0.005	[0.04, 0.22]
Sex		< 0.01	0.18	0.984	[− 0.36, 0.37]
Sex × event-related stress		0.04	0.05	0.388	[− 0.05, 0.13]
Sex × group		0.14	0.26	0.605	[− 0.38, 0.66]
Group × sex × event-related stress		− 0.10	0.06	0.112	[− 0.22, 0.02]
Social stress	4695	0.09	0.03	0.003	[0.03, 0.14]
Group		0.51	0.18	0.005	[0.16, 0.86]
Group × social stress		0.03	0.04	0.449	[− 0.04, 0.10]
Sex		0.01	0.16	0.977	[− 0.32, 0.32]
Sex × social stress		0.01	0.04	0.844	[− 0.07, 0.09]
Sex × group		0.01	0.24	0.965	[− 0.46, 0.48]
Group × sex × social stress		0.03	0.05	0.578	[− 0.07, 0.13]

Obs, number of observations; B, standardized regression coefficient; SE, standard error; CI 95%, 95% confidence interval. The dependent variable in all models is negative affect. All models control for age and lifetime depression.

**Table 3:** Estimated marginal means of stress on negative affect in the ASD and control group.

	ASD (N = 50)				Controls (N = 51)			
	Margin	SE	P	95% CI	Margin	SE	P	95% CI
**Negative affect**								
Activity-related stress	0.21	0.02	< 0.001	[0.17, 0.25]	0.10	0.02	< 0.001	[0.06, 0.14]
Event-related stress	0.21	0.02	< 0.001	[0.16, 0.25]	0.13	0.02	< 0.001	[0.08, 0.17]
Social stress	0.13	0.02	< 0.001	[0.10, 0.17]	0.09	0.02	< 0.001	[0.05, 0.13]

SE, standard error; 95% CI, 95% confidence interval; ASD, Autism Spectrum Disorder.

#### Biological stress reactivity

None of the two-way or three-way interactions reached significance (Table 4). To facilitate comparisons between studies, the estimated marginal means of the three stressors on cortisol in both the ASD and control group are presented in Supplementary Table S1.

**Table 4:** Multilevel regressions estimate of stress, group, and their interactions in the model of cortisol.

	Obs	B	SE	P	95% CI
Activity-related stress	7048	0.02	0.02	0.314	[− 0.02, 0.06]
Group		0.12	0.12	0.305	[− 0.12, 0.36]
Group × activity-related stress		− 0.01	0.02	0.566	[− 0.06, 0.03]
Sex		0.04	0.11	0.703	[− 0.18, 0.27]
Sex × activity-related stress		0.01	0.03	0.788	[− 0.04, 0.06]
Sex × group		0.10	0.16	0.551	[− 0.22, 0.41]
Group × sex × activity-related stress		− 0.03	0.04	0.433	[− 0.10, 0.04]
Event-related stress	7040	0.04	0.03	0.120	[− 0.01, 0.10]
Group		0.10	0.12	0.389	[− 0.13, 0.33]
Group × event-related stress		0.03	0.04	0.514	[− 0.05, 0.10]
Sex		0.06	0.11	0.624	[− 0.17, 0.28]
Sex × event-related stress		− 0.02	0.04	0.685	[− 0.09, 0.06]
Sex × group		0.04	0.16	0.788	[− 0.23, 0.35]
Group × sex × event-related stress		− 0.01	0.05	0.831	[− 0.11, 0.09]
Social stress	4207	0.04	0.02	0.090	[− 0.01, 0.09]
Group		0.14	0.12	0.249	[− 0.10, 0.38]
Group × social stress		− 0.05	0.03	0.152	[− 0.11, 0.02]
Sex		0.06	0.12	0.627	[− 0.17, 0.28]
Sex × social stress		− 0.02	0.03	0.660	[− 0.08, 0.05]
Sex × group		0.13	0.16	0.417	[− 0.19, 0.46]
Group × sex × social stress		− 0.02	0.04	0.639	[− 0.10, 0.06]

Obs, number of observations; B, standardized regression coefficient; SE, standard error; CI 95%, 95% confidence interval. The dependent variable in all models is CORT (i.e., log-transformed cortisol). All models were controlled for hour, hour^2^, oral contraceptive use, age, and lifetime depression.

### Sensitivity analysis

All analyses were repeated within the new sample (ASD N = 41, controls N = 51), excluding participants with depression and the use of antipsychotics. The results remained similar for all the hypotheses (see Supplementary Tables S2 and S3) .

### Exploratory analysis: the mediating effect of cortisol on stress and negative affect

Conditions for mediation were only met for event-related stress. Therefore, activity-related and social stress were excluded from further analyses. Results demonstrated a significant total effect of event-related stress on NA in the ASD group (B = 0.20, SE = 0.03, *p* < 0.001, CI [0.15, 0.25]), but no indirect effect (B < 0.01, SE < 0.01, *p* = 0.287, CI [− 0.00, 0.01]), i.e., cortisol did not mediate the association between event-related stress and NA. Moreover, there was a significant total effect of event-related stress on NA in controls (B = 0.12, SE = 0.02, *p* < 0.001, CI [0.08, 0.16]), but no indirect effect (B < 0.01, SE < 0.01, *p* = 0.057, CI [− 0.00, 0.01]). Thus, results showed a significant effect of event-related stress on NA in both groups but the association between event-related stress and NA was not mediated by cortisol levels.

## Discussion﻿

This study investigated momentary emotional and biological stress in the daily life of adults with and without ASD. A significantly stronger momentary stress reactivity in the ASD than in the control group was demonstrated, i.e., the associations between momentary NA and unpleasant events and daily activities in adults with ASD were significantly stronger than in controls, with no evidence for sex differences. Cortisol reactivity was not significantly stronger in the ASD group than in controls; associations between momentary cortisol and daily stress were neither dependent on group or sex. Lastly, no evidence for a mediating role of cortisol was found on emotional stress reactivity.

### Group and sex differences in emotional and biological stress reactivity

Adults with ASD reported more daily life activity- and event-related stress compared to controls, with both of these real-life, real-world, stressors being more strongly associated with NA in ASD. These results bridge the gap in the literature by using ESM to measure momentary emotional stress reactivity in the natural flow of daily life, multiple times a day, for a longer period. Of note, previous observational and experimental research only investigated perceived stress by using retrospective traditional questionnaires, similarly demonstrating higher stress levels in children and adults with ASD^1,6^. The current study surprisingly showed that there was no group difference in the NA increase associated with social stress even though adults with ASD reported more often that they would rather be alone than the control group. An explanation for these findings may be that adults with ASD experience the same level of social support when in the company of others^38,39^, and social support may, therefore, be a protective factor against stress^40^. There was no moderating effect of sex on emotional stress reactivity levels in both groups, such as in a previous ESM study of psychotic disorder^26^. Based on the latter study, we hypothesized a greater emotional stress reactivity in females with ASD as there is overlap between the autism and psychosis spectrum. However, the current results imply a shared stress sensitivity between males and females with ASD, which could reflect the fact that ASD is primarily a neurodevelopmental disorder that may obscure stress-related sex differences. As increased stress reactivity is associated with the emergence of diverse psychiatric disorders, the current results may comply with the absence of significant sex differences in studies on psychiatric comorbidity in ASD^41,42^. Nonetheless, future studies should aim for larger sample sizes since the current sample was relatively small to investigate sex differences.

None of the associations between the stressors and momentary cortisol levels were significantly moderated by group or sex, which although unexpected, seems consistent with some of the laboratory research showing equal cortisol responses in adults with ASD and controls^1,12^. With respect to sex differences (as mentioned above), we cannot exclude the possibility that the absence of sex differences in cortisol reactivity may be due to a lack of power, especially since there is an increased body of evidence of sex differences in cortisol reactivity^43^.

As mentioned in the introduction, well-validated stressors were used and the current methodology has previously been applied to individuals with (increased risk for) psychotic disorder^14,44^ and with 22q11.2 deletion syndrome^15^. Still, it should be noted, that cortisol response to stress is relatively slow and begins to rise within minutes of the onset of a stressful event and peaks within 20 min, with a gradual decline to baseline levels over the next hour or longer^45^. In the current study, activity-related stress and social stress were measured ‘in the moment’ and these stressors were registered at maximum 10 min after the beep signal, which also applies to the cortisol sampling. Therefore, it might be argued that the cortisol sampling occurred too early to detect changes in cortisol levels. Adding a 15–25 min time-lag between the self-report measures and the cortisol sampling could have solved this issue. Nonetheless, a recent review demonstrated that ESM studies using a 25-min lagged saliva collection versus concurrent stress assessments was equally effective^46^. A possible explanation for these findings may be that the duration of real-life stressors widely varies and that participants are often unable to report exactly when a stressful situation started or ended^47^. Thus, although the timing of cortisol measures in relation to daily stressors may be imprecise, it is possible to assess associations between these variables^47^.

Event-related stress was assessed differently because participants were asked to report an event between the previous and present beep (with an average of 90 min between each beep). Even though it is difficult to measure the duration of real-life stressors (as described above), an unpleasant event may already have happened. Therefore, some of the cortisol peaks may have been missed.

Taken together, the field could benefit from more knowledge on maximum momentary stress-cortisol cross-correlations, as was proposed by Schlotz^46^. This type of research could also gain from technological development^46^. Especially, since it is expected that wearables are going to play an important role in the next coming years^48^, e.g., to measure cortisol levels via sweat^49^. This may be less burdensome to participants compared to salivary samples, enabling researchers to study stress-cortisol correlations more easily.

In sum, the current study demonstrated a stronger emotional, but a comparable biological, stress reactivity in adults with ASD compared to controls. Current findings are in line with experimental studies that have found a significantly increased emotional response and a comparable cortisol response to stress in adults with attention-deficit/hyperactivity disorder (ADHD) relative to controls^50,51^. Because of the neurobiological and genetic overlap between ASD and ADHD^52,53^, the question may be raised whether the current findings could be explained by specific underlying mechanisms affecting stress experience and processing in individuals with neurodevelopmental disorders. Future transdiagnostic studies are needed to investigate this.

### The mediating effect of cortisol on stress and negative affect

In contrast with our expectations, cortisol did not mediate the emotional stress response, or, in other words, cortisol did not mediate the association between event-related stress and NA. We have to be careful to interpret these findings since cortisol and NA were measured at the same time. As mentioned before, event-related stress may have already happened a bit longer before the beep, which may explain why this was the only stressor meeting the conditions for mediation. Although we did not find a mediating effect of cortisol on emotional stress reactivity, the feasibility and relevance to study momentary stress responses in a naturalistic environment has been shown.

### Strengths and limitations

This is the first electronic self-monitoring study on momentary emotional and biological stress reactivity in the natural flow of daily life. The ESM, easily applicable via a mobile phone app, may have large potential for wide (clinical) usage in the autism community. Most of the participants gave positive feedback on the usage of the app and had no problems filling out the daily questionnaires. It may facilitate insight into contextualized stress experiences and other psychological experiences, shared-decision making, and enhance care-user empowerment. Another strength of this study is that multiple stressors were studied in a representative population, by including individuals using medication and with comorbid disorders. These individuals are often excluded to create a more homogeneous ASD sample and because medication may influence the cortisol response, although the generalizability of the results may become less since many adults with ASD receive some form of pharmacotherapy^54^ and they are more prone to develop comorbid disorders^55^. Therefore, we ran a sensitivity analysis excluding individuals with antipsychotic medication or current depression, which did not substantially impact the results. The ASD group was minimally treated to avoid major prior treatment effects on the stress response. Due to a relatively large number of participants and a sufficient number of self-reports and cortisol samples, it was possible to study in-group differences in cortisol levels. Nonetheless, an even larger sample would have yielded more power to the interaction analyses with sex and enable the study of subgroups because of the heterogeneity in ASD.

A well-validated social stress measure was used, which has been successfully applied in studies on individuals with depression^56^ and (clinical high risk for) psychosis^3,57^. Still, one may argue whether the preference to be alone is entirely indicative of social stress in ASD since it has been reported that children with elevated cortisol levels were more likely to engage with their peers^58^. However, another study showed that children with ASD that have the highest levels of cortisol show less social motivation^10^. Because of these contrasting findings, it may be interesting to further study the interplay between social motivation, social stress, and cortisol response in individuals with ASD.

It was a challenge to fit both the multilevel regression and the lower-level mediation models in the mediation analyses. Converge difficulties indicated that the models, with their complex random effects structure, may have been overfitted. However, solutions were found, and through a thorough comparison of different methods and programs, we are confident that the results are robust. Lastly, activity-related stress, social stress, cortisol, and NA were assessed at the same point in time. Hence, no direct causality can be inferred from these results. Thus, one could just as well assume that NA influences the subjective appraisal of activity-related stress, instead of the other way around. Either explanation, however, has clinical relevance.

## Conclusions

With respect to controls, adults with ASD showed stronger associations between momentary NA and unpleasant daily life events and activities as measured in a naturalistic environment. The associations between momentary cortisol and daily life stress were not dependent on either group or sex. The results highlight the feasibility and relevance of electronic self-monitoring in individuals with ASD, which may contribute to the development of more personalized stress-management approaches.

## Methods

### Sample

The sample included 50 participants with an ASD diagnosis (*N* = 26 males, *N* = 24 females) and 51 adults without a developmental or psychiatric disorder (*N* = 26 males, *N* = 25 females) between 18 and 65 years of age. Participants with ASD were recruited by contacting mental healthcare facilities in the South of the Netherlands, through patient associations, and via social media. The first author (KL) conducted the Autism Diagnostic Observation Schedule II^59^ module 4 (fluent speech) in all participants of the ASD group to confirm their diagnoses. Only those participants with ASD who had (i) a short-term psychological treatment history (maximum 2 years) and (ii) no past psychiatric admission were included. Medication use and other psychiatric disorders were no cause for exclusion except in the case of acute psychotic symptoms, suicidal tendencies, or bipolar disorder. The Mini-International Neuropsychiatric Interview (MINI)^60^ was used to assess the presence of psychiatric disorders in participants with ASD. The control group was recruited via social media. Participants were excluded if they had a first-degree family member diagnosed with, or suspected of having, ASD. The Autism Spectrum Quotient^61^ was used to identify the degree of ASD features in the control group; a score above 26 led to exclusion^62^. The MINI was also used to exclude any controls with a current psychiatric disorder. General exclusion criteria were (i) suffering from known genetic abnormalities, brain injury, epilepsy, or metabolic disorders, and (ii) an intelligence quotient (IQ) below 70. The latter was screened with two subtests (matrix reasoning and vocabulary) of the Wechsler Adult Intelligence Scale—Fourth Edition^63^.

### Procedure

This study was approved by the medical ethics committee of Maastricht University (NL51997.068.15) and was carried out in accordance with the Declaration of Helsinki^55^. All participants were well informed about the study and gave written informed consent before the first appointment. Of note, we recruited a group of adults with ASD without a co-occurring intellectual disability. There were no concerns regarding their mental competence or decision-making capacity. Hence, they were capable to give written informed consent. During the first appointment, participants were screened for meeting the inclusion criteria. The ESM protocol and the collection of the salivary samples were explained in the following session.

#### The experience sampling method

As reported in another paper on this sample^64^, daily life assessments were done with the ESM, delivered via the PsyMate application. Participants received an iPod or downloaded the app on their smartphone. During 10 days, 10 times a day, the application sent an alert at random moments between 07:30 h and 22:30 h. Participants then answered questions about mood, social context, and activities, completing their reports within an allotment of 10 min after the signal. The questionnaire consisted of 7-point Likert scales to capture momentary experiences and categorical questions to capture context (e.g., social context, activities). Participants were encouraged to follow their daily routines. All participants were contacted by telephone after 2 days of sampling to ask if they experienced any problems concerning the protocol. It was also possible for them to contact the researchers if they had questions or experienced problems with the ESM data collection. Exclusion from the analysis followed in case less than 30% valid reports were acquired (30 out of 100), as previous work has shown that these data are less reliable^65^.

#### Cortisol sampling

In line with previous studies^13–15^ participants were asked to take a saliva sample (to measure cortisol) using cotton swabs within a maximum time-frame of 10 min after signaling of the PsyMate application. Thus, when there was a beep signal, the participant took a cotton salivette (from a plastic tube) and placed it in his/her mouth. After this, the cotton salivette was put back in the plastic tube, and the participants were asked to write down the time on the tube. All saliva samples were placed in a freezer at the home of the participant until the debriefing session. In most cases, the debriefing session was scheduled within a few days after the completion of the ESM protocol.

After collecting the data, participants were invited for a debriefing session and their experiences were evaluated.

### Measures

#### Momentary stress

As described in a recent publication on this sample^64^, stress was conceptualized as subjectively appraised stress after regular daily life encounters or activities. Three different stress measures were obtained: activity-related, event-related, and social stress.

*Activity-related stress* was operationalized, starting with the question “What are you doing?”. Three items followed this question, i.e., “I would rather do something else”; “This is difficult for me” and “I can do this well”, reverse coded. These questions were scored on 7-point Likert scales (1 = not, 7 = very) and were combined into a mean activity-related stress variable.

*Event-related stress* was based on the question “What was the most important event since the last beep?”. Participants subsequently scored how pleasant/unpleasant the event was on a bipolar scale (− 3 very unpleasant, 0 neutral, + 3 very pleasant). Positive events (scores 1, 2, and 3) were recorded to zero, and negative scores were reverse coded (i.e., higher ratings reflect more stress).

*Social stress* was operationalized by asking participants if they were in the company of others or alone. If in the company of others, they were asked to rate the item “I would prefer to be alone” (1 = not, 7 = very).

#### Negative affect

The choice for the NA items in this study was guided by the extensive previous ESM literature using the same construct of NA (e.g.,^57,66,67^). These items were originally based on the Positive and Negative Affect Schedule (PANAS)^68^. More specifically, affect was measured with the items (“I feel ….”) down, insecure, lonely, anxious, irritated, relaxed, enthusiastic, satisfied, and cheerful. All items were rated on 7-point Likert scales (1 = not, 7 = very). Factor analyses showed that these items loaded on two factors: NA and positive affect. The items down, insecure, lonely, anxious, and irritated loaded on the NA factor. Irritated, however, also had high cross-loadings on the positive affect factor. Therefore, the mean of the four items down, insecure, lonely, and anxious was used as a measure of NA in the analyses.

#### Momentary cortisol

After collection, the samples were stored in a freezer (− 20 °C) at Maastricht University. At a later stage, the samples were sent by courier to Dresden Lab Service GmbH (Dresden, Germany) to be assayed. Cortisol levels were determined in duplicate using a time-resolved immunoassay with a fluorescence detector^69^. Samples with cortisol > 44 nmol/L were excluded (N = 3) from the statistical analyses. Raw cortisol values were log-transformed to reduce the skewness of their distribution, generating the variable CORT.

### Statistical analyses

All analyses were carried out in Stata version 13.1^70^ and R^71^. ESM data have a multilevel structure. Therefore, two-level mixed-effects regression models (using the 'mixed' command in Stata) were used to analyze the ESM data, with observations (level 1) nested within-subjects (level 2). The independent variables, their interactions, and the covariates were entered into the models as fixed effects. Random intercepts and random slopes were added at the subject level, using an unstructured covariance matrix for the random effects. Models were fitted using restricted maximum likelihood estimation (REML). Fixed effects were tested via Wald-type tests with α = 0.05 (two-sided). As a first step, separate multilevel models were fitted to test whether the levels of NA, momentary stress, and CORT (dependent variables) differed between groups (independent variable; 0 = controls, 1 = ASD).

#### Group and sex differences in emotional and biological stress reactivity

Models were fitted for each type of appraised stress (activity-related, event-related, and social stress) as a continuous predictor and NA or CORT as the outcome variable. Age and lifetime depression (yes/no) were examined as covariates in all models, and oral contraceptive use (yes/no) was added in all models involving CORT. Time of the day ('hour') and its square ('hour^2^') were included as predictors in all analyses regarding cortisol to model the diurnal cortisol curve. The 'hour' variable was centered at 15:00 h to reduce collinearity with its squared value. Two-way (stress × group, stress × sex, group × sex) and three-way (stress × group × sex) interactions were used to test whether associations between stress and NA or CORT differed by group or sex. Based on each fitted model, we computed the slopes (of stress on NA or CORT) for all four groups with corresponding 95% confidence intervals (CIs). In case of a significant three-way interaction, pairwise differences were computed between the simple slopes to investigate the effect of both group and sex in the association between stress and NA or CORT. In case of only a significant two-way interaction, the simple slopes for the associations between stress and NA were calculated (command: margins). Regarding the sample size (*N* = 101), simulation papers demonstrated that 100 subjects were sufficient to investigate two-way interactions^72,73^, and with a minimum of 30 reports for each subject, it was possible to detect either small, medium, and large effect sizes^73^. However, it was expected that the current sample size yielded limited power to investigate a three-way interaction^74^.

#### Sensitivity analysis

To verify whether the results of the primary analyses were robust, we performed a sensitivity analysis. We excluded those with depression because depression is known to be associated with perceived stress^75^ and NA^76^. There is also evidence that medication use may impact the emotional and biological stress response^77^. A priori analyses on this sample demonstrated that antipsychotic medication was a significant covariate in the models of NA and CORT: antidepressants, anxiety, and insomnia medication were not. Therefore, those using antipsychotics were excluded from the analyses. This led to the exclusion of 9 participants from the ASD group (current depression *n* = 3, antipsychotic use *n* = 6), and none in the control group.

#### Exploratory analysis: the mediating effect of cortisol on stress and negative affect

First, it was explored whether conditions for mediation were met. That is, we verified, in separate regression models, that the independent variable (each stress measure), the mediator (CORT), and the dependent variable (NA) were all significantly associated with each other. When conditions for mediation were met, a lower-level mediation analysis to estimate the indirect (i.e., mediated), total, and the direct effect was carried out. We ran lower-level (i.e., within-person) mediation models^78^ using the lmer function from the R package lme4^79^. Stress, NA, and CORT were centered at the person mean, removing all between-subject effects^80^, with hour and hour^2^ as covariates. The model included random intercepts and slopes for the three different paths of the mediation model, and error variances were allowed to differ across the equations for the mediator as an outcome and Y as an outcome; optimx method 'nmkb' was used as an optimizer. The bootmlm package (using vcov_vc) was used to get the covariance matrix for the random effects from which the covariance between the X→mediator and the mediator→Y path was extracted. Finally, random indirect and random direct effects were calculated from these estimates using the equations in Bauer et al.^78^.

## Supplementary Information

Supplementary Information.

## Data Availability

The datasets for this manuscript are not publicly available due to patient confidentiality and participant privacy. Requests to access the datasets should be directed to Machteld Marcelis, m.marcelis@maastrichtuniversity.nl.
