# Supplementary material for: A momentary assessment study on emotional and biological stress in adult males and females with autism spectrum disorder
Source: Sci Rep. 2021 Jul 8;11:14160. doi: 10.1038/s41598-021-93159-y (PMC8266874; doi:10.1038/s41598-021-93159-y)
Supplement: Supplementary file 1 — Supplementary Information. [file 41598_2021_93159_MOESM1_ESM.docx]

**A momentary assessment study on emotional and biological stress in adult males and females with autism spectrum disorder**

Kim van der Linden, MSc^1,2,*^, Claudia Simons, PhD^1,2^, Wolfgang Viechtbauer, PhD^2^, Emmy Ottenheijm, MSc^1^, Thérèse van Amelsvoort MD PhD^2^, Machteld Marcelis MD PhD^1,2^

1. GGzE, Mental Health Institute Eindhoven, Eindhoven, The Netherlands

2. Department of Psychiatry and Neuropsychology, School for Mental Health and Neuroscience (MHeNS), Maastricht University, Maastricht, The Netherlands

**Supplementary material**

**Table S1. Estimated marginal means of stress on cortisol in the ASD and control group**

|  |  |  | **ASD (N = 50)** | | | |  |  | **Controls (N = 51)** | | | |  |
| --- | --- | --- | --- | --- | --- | --- | --- | --- | --- | --- | --- | --- | --- |
|  | **Margin** | | | **SE** | **P** | **95% CI** | | **Margin** | | **SE** | **P** | **95% CI** | |
| Activity-related stress | -.01 | | | .01 | .646 | [-.03, .02] | | .02 | | .01 | .091 | [-.00, .05] | |
| Event-related stress | .06 | | | .02 | .001 | [.02, .09] | | .04 | | .02 | .055 | [-.00, .07] | |
| Social stress | -.02 | | | .01 | .133 | [-.05, .01] | | .03 | | .02 | .040 | [.00, .07] | |

SE, standard error; 95% CI, 95% confidence interval; ASD, Autism Spectrum Disorder

**Table S2. Sensitivity analysis: multilevel regressions estimate of stress, group, sex, and their interactions in the model of negative affect**

|  | **Obs** | **B** | **SE** | **P** | **95% CI** |
| --- | --- | --- | --- | --- | --- |
|  |  |  |  |  |  |
| Activity-related stress | 7116 | .09 | .03 | .002 | [.03, .15] |
| Group |  | .41 | .15 | .005 | [.13, .70] |
| Group x activity-related stress |  | .09 | .04 | .033 | [.01, .18] |
| Sex |  | -.00 | .13 | .996 | [-.25, .25] |
| Sex x activity-related stress |  | .01 | .04 | .750 | [-.07, .09] |
| Sex x group |  | -.02 | .19 | .913 | [-.40, .36] |
| Group x sex x activity-related stress |  | .02 | .06 | .792 | [-.10, .14] |
|  |  |  |  |  |  |
| Event-related stress | 7108 | .11 | .03 | .002 | [.04, .17] |
| Group |  | .53 | .19 | .005 | [.16, .90] |
| Group x event-related stress |  | .12 | .05 | .022 | [.02, .22] |
| Sex |  | .02 | .16 | .913 | [-.30, .34] |
| Sex x event-related stress |  | .04 | .05 | .385 | [-.05, .13] |
| Sex x group |  | .14 | .25 | .572 | [-.35, .63] |
| Group x sex x event-related stress |  | -.09 | .07 | .201 | [-.22, .05] |
|  |  |  |  |  |  |
| Social stress | 4353 | .09 | .03 | .002 | [.03, .14] |
| Group |  | .45 | .18 | .014 | [.09, .80] |
| Group x social stress |  | .02 | .04 | .605 | [-.06, .10] |
| Sex |  | .01 | .16 | .931 | [-.29, .32] |
| Sex x social stress |  | .01 | .04 | .858 | [-.07, .08] |
| Sex x group |  | .07 | .24 | .767 | [-.40, .54] |
| Group x sex x social stress |  | .02 | .05 | .731 | [-.09, .12] |

Obs, number of observations; B, standardized regression coefficient; SE, standard error; CI 95%, 95% confidence interval. The dependent variable in all models is negative affect. All models control for age and lifetime depression.

**Table S3. Sensitivity analysis: multilevel regressions estimate of stress, group, and their interactions in the model of cortisol**

|  | **Obs** | **B** | **SE** | **P** | **95% CI** |
| --- | --- | --- | --- | --- | --- |
|  |  |  |  |  |  |
| Activity-related stress | 6392 | .02 | .02 | .321 | [-.02, .06] |
| Group |  | .18 | .11 | .094 | [-.03, .40] |
| Group x activity-related stress |  | -.01 | .03 | .609 | [-.07, .04] |
| Sex |  | .04 | .10 | .696 | [-.16, .24] |
| Sex x activity-related stress |  | .01 | .03 | .778 | [-.04, .06] |
| Sex x group |  | .03 | .15 | .817 | [-.25, .32] |
| Group x sex x activity-related stress |  | -.03 | .04 | .394 | [-.10, .04] |
|  |  |  |  |  |  |
| Event-related stress | 6384 | .04 | .03 | .123 | [-.01, .10] |
| Group |  | .16 | .11 | .142 | [-.05, .38] |
| Group x event-related stress |  | .06 | .04 | .180 | [-.03, .14] |
| Sex |  | .05 | .10 | .597 | [-.14, .25] |
| Sex x event-related stress |  | -.02 | .04 | .678 | [-.09, .06] |
| Sex x group |  | -.02 | .14 | .868 | [-.31, .26] |
| Group x sex x event-related stress |  | -.04 | .05 | .482 | [-.14, .07] |
|  |  |  |  |  |  |
| Social stress | 3903 | .04 | .02 | .071 | [-.00, .09] |
| Group |  | .20 | .12 | .092 | [-.03, .44] |
| Group x social stress |  | -.04 | .03 | .190 | [-.11, .02] |
| Sex |  | .05 | .11 | .611 | [-.15, .26] |
| Sex x social stress |  | -.02 | .03 | .658 | [-.08, .05] |
| Sex x group |  | .06 | .16 | .693 | [-.25, .38] |
| Group x sex x social stress |  | -.03 | .05 | .529 | [-.12, .06] |

Obs, number of observations; B, standardized regression coefficient; SE, standard error; CI 95%, 95% confidence interval. The dependent variable in all models is CORT (i.e., log-transformed cortisol). All models were controlled for hour, hour^2^, oral contraceptive use, age, and lifetime depression.
